# A Comparison Between the Growth of Naturally Occurring Three-Dimensional Cracks in Scalmalloy^®^ and Pre-Corroded 7085-T7452 and Its Implications for Additively Manufactured Limited-Life Replacement Parts

**DOI:** 10.3390/ma18245586

**Published:** 2025-12-12

**Authors:** Daren Peng, Shareen S. L. Chan, Ben Main, Andrew S. M. Ang, Nam Phan, Michael R. Brindza, Rhys Jones

**Affiliations:** 1 ARC Industrial Transformation Training Centre on Surface Engineering for Advanced Materials, School of Engineering, Swinburne University of Technology, John Street, Melbourne, VIC 3122, Australia; daren.peng@monash.edu (D.P.); slchan@swin.edu.au (S.S.L.C.); aang@swin.edu.au (A.S.M.A.); 2 Department of Mechanical and Aerospace Engineering, Monash University, Melbourne, VIC 3800, Australia; 3 Defence Science and Technology Group, 506 Lorimer Street, Melbourne, VIC 3207, Australia; 4 Structures Division, Naval Air Systems Command, Patuxent River, MD 20670, USA; 5 Air Warfare & Weapons Department/Air Platforms Division, Office of Naval Research, Arlington, VA 22217, USA

**Keywords:** corrosion pits, 7085-T7452, Scalmalloy^®^, fatigue crack growth, limited-life replacement parts

## Abstract

This paper is the first to reveal that the conventionally built aluminium alloy (AA) 7085-T7452 has mechanical properties, viz: a yield stress, ultimate strength, and an elongation to failure, that are similar to that of laser powder bed fusion (LPBF) built Scalmalloy^®^. Following this observation, the growth of cracks that nucleated from corrosion pits in AA7085-T7452 specimens that had been exposed to a 5 wt% NaCl salt fog environment at 35 °C according to ASTM B117-19 standard for fourteen days is then studied. The specimen geometries were chosen to be identical to those associated with a similar study on Boeing Space, Intelligence, and Weapon Systems (BSI&WS) LPBF built Scalmalloy^®^. This level of prior exposure led to pits in AA7085-T7452 that were approximately 0.5 mm deep with a surface width/diameter of up to approximately 1.5 mm. These pit sizes are broadly consistent with those leading to fatigue crack growth (FCG) in AA 7050-T7451 structural parts on the RAAF F/A-18 Classic Hornet fleet operating in a highly corrosive environment. Fatigue tests on these AA7085-T7452 specimens, under the same spectrum as used in the BSI&WS LPBF Scalmalloy^®^ study, reveals that AA7085-T7452 and Scalmalloy^®^ have similar crack growth histories. This, in turn, leads to the discovery that the growth of naturally occurring three-dimensional (3D) cracks in AA 7085-T7452 could be predicted using the crack growth equation developed for BSI&WS LPBF Scalmalloy^®^, albeit with allowance made for their different fracture toughness’s. These findings suggest that Scalmalloy^®^ may be suitable for printing parts for both current and future attritable aircraft.

## 1. Introduction

There are several reasons for choosing to investigate the effect of pre-exposure to an aggressive environment on the growth of naturally occurring three dimensional (3D) cracks in AA7085-T7452, viz:

(i) It has primary structural applications in both current commercial and military aircraft, see Main et al. [1].
(ii) European Aviation Safety Authority (EASA) Safety Information Bulletin (SIB) 2018-04R2 [2] revealed that AA7085-T7452 airframes can experience environmentally assisted cracking (EAC) issues.
(iii) As stated in Appendix X3 of the ASTM fatigue test standard ASTM E647 [3]: “Fatigue cracks of relevance to many structural applications are often small or short for a significant fraction of the structural life.”
(iv) As explained in MIL-STD-1530Dc [4], which addresses the airworthiness certification of conventionally built metallic airframes, and in United States Air Force (USAF) Structures Bulletin EZ-SB-19-01 [5], which addresses AM parts, the airworthiness certification of both conventionally and additively manufactured aircraft parts requires a durability assessment which, as also stated, is best done using a linear elastic fracture mechanics (LEFM) based approach. Furthermore, as explained in the USAF F-15 study reported in [6], this requires using a valid small crack *da/dN* versus ∆*K* curve. Here ∆*K* = *K_max_* − *K_min_*, where *K_max_* and *K_min_* are the maximum and minimum values of the stress intensity factor (*K*) in a cycle.

At this point it should also be stressed that the focus of this paper is on the fracture mechanics needed to perform the durability assessment mandated in USF MIL-STD-1530D [4] rather than on materials science related aspects.

In this context, it should be noted that although EAC in Al-Zn-Mg-Cu aluminium alloys has long been studied, see Holroyd [7], EASA SIB 2018-04R2 [2] was arguably the first to report in-service EAC issues associated with AA 7085-T7452. These issues arose as a result of aircraft spending extended periods of time on the ground, for more details see [2]. These (AA7085-T7452) fleet findings and the EASA bulletin [2], led to several subsequent studies into the effect of an aggressive environment on the corrosion and stress corrosion cracking of AA7085-T7452 as well as on the effect of environmental degradation on crack growth in AA7085-T7452 [1,8,9,10,11,12,13,14,15,16,17,18,19]. This, in turn, led to a November 2024 joint United States Air Force Research Laboratory (AFRL) and United States Federal Aviation Administration (FAA) Technical Interchange Meeting [20] that focused specifically on the issue of environmental-assisted cracking (EAC) associated with high-strength 7XXX series aluminium alloys. Of the various presentations contained in [20], those of Waite and Passard [21], Barrett [22], and Yates [23], which present the status as perceived by the EASA, Airbus and Lockheed respectively, are perhaps the most relevant to the current paper. The papers by Waite and Passard [21] and Yates [23] were particularly interesting in that they flagged instances of EAC in AA7085-T7452 parts in both military and civilian aircraft. The paper by En Nami et al. [24] is also interesting in that it has implication for minimizing corrosion damage at intersection of a fastener hole and the surface.

**Figure 1:**
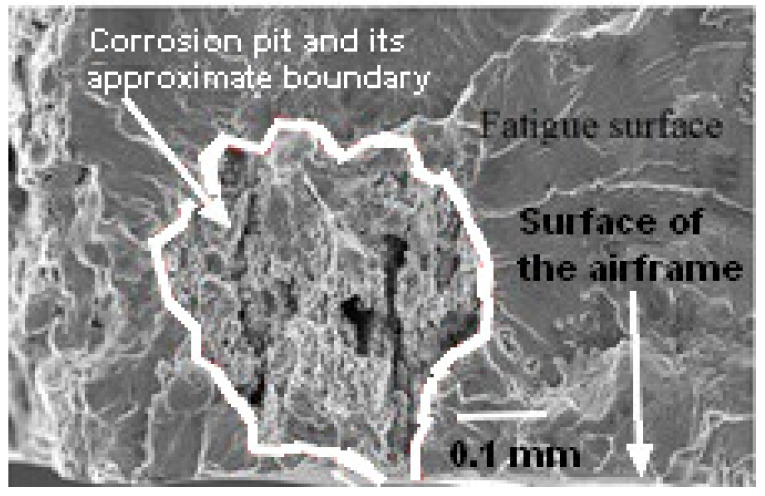
Quantitative fractography of a measured corrosion pit in a RAAF F/A-18 A/B service aircraft.

In this context, it should be noted that it has long been known that the maintenance costs and loss of aircraft availability due to environmental degradation-related issues are particularly significant, see [25]. The extent of corrosion damage to aluminium alloy (AA) parts in US Navy aircraft is aptly highlighted by Mendoza [26] and Shipilov [27]. Examples of typicalncorrosion pits in Royal Australian Air Force (RAAF) F/A-18 aircraft, from which cracks nucleated, are given in [28,29,30] where it was found that the depths of the pits ranged from approximately 0.2 mm to approximately 0.44 mm. One such (typical) corrosion pit is shown in Figure 1. In this instance the depth of the corrosion pit is approximately 0.3 mm. The importance of managing corrosion maintenance costs and the effect of corrosion on aircraft availability is also highlighted in Molent and Wanhill [28]. Numerous examples of the fact that extensive corrosion occurs even for parts that have been anodised or have chromate-based protection coatings are given in Mendoza [26]. That said, and noting that Yates [23] reported the problem of environmental damage at fastener holes in anodized AA7085-T7452, to the best of the author’s knowledge there is currently no study in the open literature on the effect of prior exposure to an aggressive environment on the growth of naturally occurring 3D cracks that nucleate at the intersection of the bore of a fastener hole and the anodised surface of AA7085-T7452. Furthermore, there is no study in the open literature where the crack growth history for such problems is predicted. This is despite a predictive capability being mandated in MIL-STD-1530D [4]. Consequently, one objective of the current paper is to address this shortcoming.

Having discussed why investigate naturally occurring 3D cracks in AA7085-T7452, let us address the following questions:

(i) Why compare naturally occurring 3D cracks in AA7085-T7452 with naturally occurring 3D cracks in BSI&WS LPBF built Scalmalloy^®^?
(ii) Why raise the implications of this study for “limited-life” AM parts?

The answer to the first question is that the authors had previously [31,32,33] studied the growth of both long cracks and naturally occurring 3D cracks in BSI&WS LPBF built Scalmalloy^®^. As a result, the test program on naturally occurring 3D cracks in AA7085-T7452 led to the realization that the crack growth equation governing the growth of naturally occurring 3D cracks in AA7085-T7452 was essentially the same, albeit allowing for small differences in the cyclic fracture toughness.

The answer to the second question follows from the following observations:

(a) That the previous study [31] revealed that BSI&WS LPBF built Scalmalloy^®^ is more damage tolerant than conventionally built AA7075-T6, which is used in a variety of both fixed and rotary wing military and civil aircraft;
(b) That BSI&WS LPBF Scalmalloy^®^ is particularly resistant to corrosion [33], the materials science explanation for this is given in [33]; that the durability of BSI&WS LPBF Scalmalloy^®^ is predictable [32,33]. (Other studies that highlight Scalmalloy’s^®^ excellent resistance to corrosion can be found in [34,35,36].);
(c) That BSI&WS LPBF built Scalmalloy^®^ has mechanical properties that are equivalent to that of conventionally manufactured AA 7075-T6 and superior to those of the conventionally manufactured AA 2024-T3 [31].
(d) That the 2019 US Department of Defense (DoD) Memo [37] mandates that AM will be used to “increase logistics resiliency, and improve self-sustainment”;
(e) That USAF Structures Bulletin EZ-SB-19-01 [5] subsequently stated that the most difficult challenge facing the airworthiness certification of an AM part is to establish an “accurate prediction of structural performance” specific to its durability and damage tolerance (DADT);
(f) That USAF Structures Bulletin EZ-SB-19-01 [5] clearly stated that one of the primary considerations for a limited-life AM part is its durability;
(g) That Muhammad et al. [38] concluded that of all the AM aluminium alloys studied Scalmalloy^®^ had superior tensile strength, Young’s modulus, yield strength, and elongation to failure;
(h) That, although not previously reported, AA7085-T7452 and LPBF Scalmalloy^®^ have similar mechanical properties, see Table 1.
(i) That NASA [39] have proposed an approach to the certification of AM parts that which is based on ‘material equivalence’.

Here it should be noted that the definition of a limited life replacement part is one that, although its life is less than the design life of the ‘original’ wrought part, it offers the potential to ensure continued operational usage for an interim period, at least until a new part can be obtained. Such AM limited life replacement parts would meet the intent of the US DoD Memo [37] to “increase logistics resiliency, and improve self-sustainment”.

**Table 1:** Values of σ_y_, σ_ult,_ and strain to failure of both die forged AA7085-T7452 and Scalmalloy^®^.

	σ_y_ (MPa)	σ_ult_ (MPa)	Strain to Failure (mm/mm)
LPBF Scalmalloy^®^, heat treated at 325 °C for 4 h, from Muhammad et al. [38].	508	530	0.16
AA 7085-T7452, values as given by SAE International [40]	448–462	496–503	0.07–0.10

As a result, it would appear that BSI&WS LPBF built Scalmalloy^®^ may have the potential to build limited-life replacement parts for both AA 7075-T7xxx and AA 2024-T3 parts for military aircraft and for the printing of limited-life parts for attritable aircraft. (For more details on, as well as the definition of attritable aircraft see Colombi et al. [41] and Hayes [42]. Examples of current attritable aircraft include the Boeing MQ-28A Ghost Bat, Kratos XQ-58 Valkyrie and the Bayraktar Kızılelma.) This potential may be easier to be realised if the NASA approach to the certification of AM parts [39], which is based on ‘material equivalence’, becomes accepted.

Unfortunately, as is evident in the above discussion, BSI&WS LPBF Scalmalloy^®^ has (currently) only been compared to what are sometimes termed ‘legacy’ aluminium alloys, namely AA 7075-T6 and AA 2024-T3. What is needed is a comparison between Scalmalloy^®^ and more modern aluminium alloys such as 7050-T7451 and 7085-T7452. Consequently, the purpose of this paper is fourfold, viz:

(1) To compare the corrosion seen by identical AA7085-T7452 and BSI&WS LPBF Scalmalloy^®^ when placed in the same ASTM B117-19 environmental chamber [43] and subjected to the same environmental conditions.
(2) To highlight the similarity between the growth of naturally occurring 3D cracks in identical BSI&WS LPBF Scalmalloy^®^ and AA7085-T7452 specimens when subjected to the same variable amplitude load spectrum.
(3) To use this discovery to estimate the crack growth equation associated with naturally occurring 3D cracks in pre-exposed AA7085-T7452, and to then use this equation to predict their growth.
(4) To use this equation to predict the growth of cracks in an anodised pre-exposed AA7085-T7452 specimen with a fastener hole which has corrosion damage at the intersection between the bore of the hole and the anodised surface.

This raises the question of how to create “natural cracks” in AA7085-T7452. Fortunately, as discussed in [44,45] and as shown in Figure 1, it has long been known that corrosion pits can nucleate cracks that will grow in operational service. Consequently, since:

(a) the crack growth histories associated with BSI&WS LPBF Scalmalloy^®^ specimens that had been exposed for twenty-eight days to a 5 wt% salt fog environmental chamber at 35 °C according to ASTM B117-19 standard, as well as with BSI&WS LPBF Scalmalloy^®^ that had not been pre-exposed, are known [32,33];
(b) the crack growth equation governing these BSI&WS LPBF Scalmalloy^®^ tests is known [32,33];

The intention was that the test program outlined above would generate the natural 3D cracks needed for this study. An additional reason for adopting this this test protocol, i.e., prior exposure to an aggressive environment followed by fatigue testing, was that Molent and Wanhill [28], Barter and Molent [29,30], Main et al. [44], Molent [45], Trathan [46], Jones [47], Chen [48] and Barter et al. [49] have suggested that corrosion and fatigue in operational airframes often decouple, with corrosion being associated with an aircraft’s time on the ground and fatigue being associated with in-flight loads. If this supposition is true then the information obtained from this test protocol would be particularly relevant to operational aircraft.

As previously mentioned, and as stressed by Lincoln et al. [6], valid small and near threshold fatigue crack growth rate (FCGR) *da/dN* versus Δ*K* data is essential for the prediction of the economic life of an airframe. This statement also holds when attempting to predict/correlate fatigue analysis with cracks that nucleate from corrosion pits. Unfortunately, with the exception of the studies by Main et al. [1] and Dixon et al. [50], there are few studies that have measured the FCGRs of small or short cracks in AA7085-T7452. The majority of current studies [15,51,52] either examine long fatigue cracks using test geometries and methods specified in the main body of the fatigue test standard ASTM E647 [3]. Unfortunately, as explained in Appendix X3 of ASTM E647, crack growth data obtained using these test methods are not applicable for assessing in-service cracking.

Consequently, in addition to enabling a direct comparison with that of BSI&WS LPBF built Scalmalloy^®^, an objective of this paper is to attempt to help alleviate this shortcoming by examining the effect of corrosion pitting that results from the prior exposure of AA7085-T7452 in an ASTM B117-19 5 wt% NaCl salt fog at 35 °C environment for fourteen days on the growth of naturally occurring 3D cracks. This time scale was chosen since it will be shown to result in corrosion pit depths and surface widths in the AA7085-T7452 specimens, that are similar to that reported in Barter et al. [49] and Main et al. [53] for AA7050-T7451 parts on RAAF F/A-18 A/B Classic Hornet that had seen prolonged downtime in an aggressive coastal environment, as well as that reported by Barter et al. [30] for a F/A-18 A/B bulkhead that had been shot peened and left outdoors for an extended period of time.

## 2. Materials and Methods

### 2.1. Pre-Exposure to an ASTM B117-19 5 wt% at 35 °C Environment

In order to achieve the goals stated in the Introduction, three distinct series of tests on AA7085-T7452 specimens are required. The first test program involves plain dogbone specimens that have not been anodized and which have identical geometries to the BSI&WS LPBF Scalmalloy^®^ specimen tests performed in [32,33].

**Figure 2:**
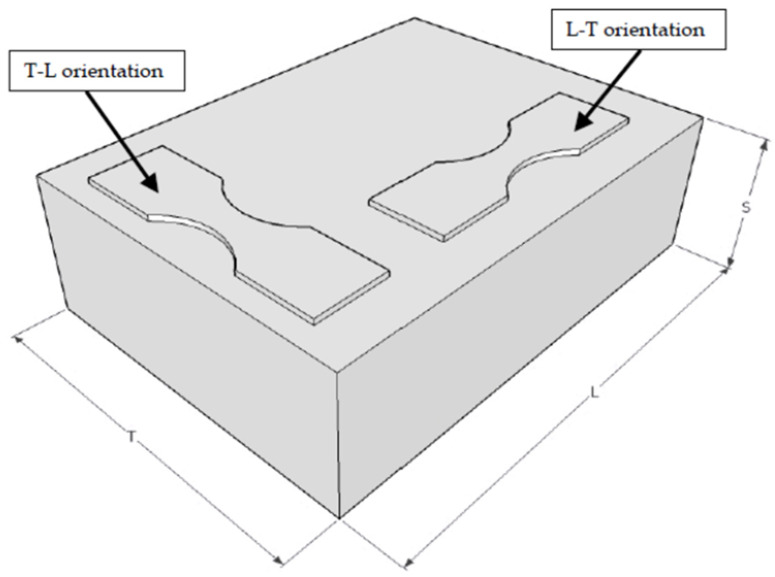
Schematic diagram showing the orientation of T-L specimens manufactured from the forging.

**Figure 3:**
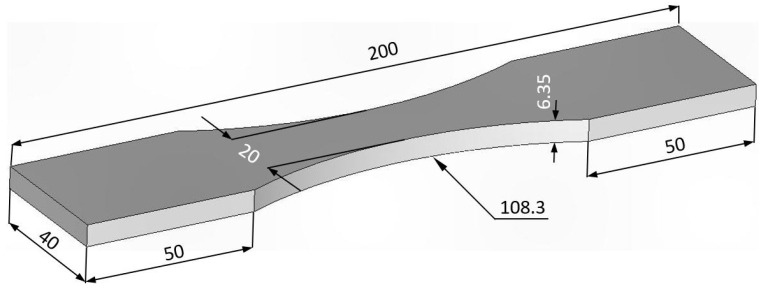
Geometry of the base-line 7085_T7452 test specimens, all dimensions are in mm.

The AA7085-T7452 specimens used in this (first) test program were machined from an AMS 4414 compliant AA7085-T7452 die forging in the T-L orientation as per Figure 2. The geometry of these specimens, which are referred to as base-line 7085-T7452 specimens, is shown in Figure 3. This geometry was chosen because it coincides with that of the BSI&WS LPBF Scalmalloy^®^ specimens tested in [32,33].

In this test program the AA7085-T7452 specimens were exposed to the same ASTM B117-19 environment, viz: the same 5 wt% NaCl salt fog at 35 °C, as were the Scalmalloy^®^ specimens tested in [33]. This was done to enable a direct comparison between the resistance of AA7085-T7452 and BSI&WS LPBF Scalmalloy^®^ to environmental degradation. It was also used to create corrosion pits, from which cracks can nucleate and subsequently grow.

Here is should be noted that studies into the effect of exposure on AA7085-T7452 for extended periods in actual marine environments, as well as in high-humidity air and in salt fog environments, such as that outlined in ASTM B117-19, can be found in the papers by Yang et al. [8], Yuang et al. [9], Schwarzenböck et al. [10], Prabhu [11], Free et al. [12], Tao et al. [13] and Shi et al. [14]. These various references present both the materials science, electrochemistry and the mechanisms associated with corrosion pitting in AA7085-T7452. As such, these various materials science related topics will not be discussed in any detail in the present paper, rather the focus shall be on comparing the extent of corrosion damage in AA7085-T7452 with that seen in tests on BSI&WS LPBF Scalmalloy^®^ and on the (subsequent) growth of fatigue cracks that nucleate from corrosion pits in AA7085-T7452.

### 2.2. The Fatigue Test Program on Pre-Exposed Base-Line AA 7085-T7452 Specimens

On completion of the first test program the pre-exposed AA7085-T7452 specimens were then fatigue tested. The marker block load spectrum applied to these AA7085-T7452 specimens, which were oriented in the TL direction, consisted of: 10,000 cycles at *R* = 0.8 and 300 cycles at *R* = 0.1. The maximum load P_max_ in each load block was held constant at 32 kN. The test frequency was 5 Hz. This corresponds to a maximum stress, in the working section, of approximately 252 MPa. This load spectrum was chosen since it was also used in the fatigue tests reported in [33] for BSI&WS LPBF Scalmalloy^®^ specimens.

This marker block load spectrum enabled the crack growth histories of the various naturally occurring 3D cracks that nucleated and subsequently grew in these AA7085-T7452 tests to measured. This in-turn enabled the crack growth histories associated with naturally occurring 3D cracks in both materials to be compared. This comparison was important since it enabled the equation governing the growth of naturally occurring cracks in AA7085-T7452 to be estimated. This estimated crack growth equation was then substantiated by using it to compute the crack growth histories in these tests. This estimated crack growth equation was then further substantiated by using it to compute the crack growth histories in these tests as well as in a subsequent test on an anodised AA7085-T7452 specimen with a fastener hole, see Section 2.3.

### 2.3. The Third AA7085-T7452 Test Program—An Anodised Specimen with a Fastener Hole

**Figure 4:**
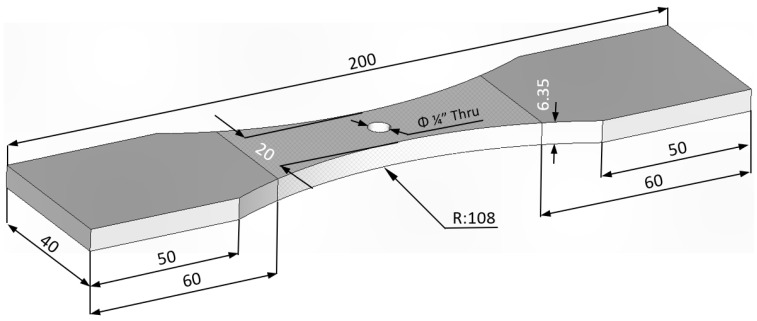
Geometry of the durability test specimen, all dimensions are in mm.

The third test program used a similar dogbone specimen geometry to that shown in Figure 3. The difference was that this specimen contained a ¼ inch (6.35) centrally located hole, see Figure 4. The specimen was anodized prior to the hole being drilled, see Figure 4. After the fastener hole was drilled, the specimen was exposed to the same ASTM B117-19 environment as that discussed above, viz: a 5 wt% NaCl salt fog at 35 °C.

The load spectrum applied to this now pre-corroded AA7085-T7452 specimen consisted of three different repeated marker load blocks. In each of these repeated load blocks the load was held constant and the spectrum consisted of 15,000 cycles at *R* = 0.8 and 300 cycles at *R* = 0.1. In other words, the marker load blocks are scaled versions of each other. For convenience, the three load spectra are named as load Spectrum 1, load Spectrum 2 and load Spectrum 3. Spectrum 1 had a maximum load in the spectrum of 10.8 kN. It’s purpose was to sharpen any cracks that developed. Fractography subsequently revealed that two diametrically cracks developed. The maximum value of the load in Spectrum 2 was 13.0 kN. Fractography revealed at the beginning of Spectrum 2 the surface crack length associated with Crack 1 was approximately 0.34 mm. The maximum value of the load in Spectrum 3 was 15.17 kN. Fractography revealed at the beginning of Spectrum 3 the surface crack length associated with Crack 1 was approximately 1.5 mm. The various load spectra were chosen so as to shorten the duration of the test.

The measured crack growth histories associated with this (third) test program are predicted using the crack growth equation determined in the second test program, i.e., in Section 2.2. The purpose of this test program is therefore two-fold, viz:

(i) To highlight that, as has been seen in operational aircraft [26], corrosion can arise at fastener holes even if the surfaces of the AA7085-T7452 have been anodized.
(ii) To investigate if the crack growth equation developed in the previous test program for naturally occurring 3D cracks that emanate from corrosion damage, can be used to predict the growth of cracks that initiate in an anodised AA7085-T7452 specimen with a fastener hole that has been pre-corroded in an ASTM B117-19 5 wt% salt fog at 35 °C.

### 2.4. The Crack Growth Analyses

As will be subsequently shown, these tests revealed that the growth of naturally occurring cracks in precorroded AA7085-T7452 is very similar to that of naturally occurring 3D cracks in BSI&WS LPBF AM Scalmalloy^®^. Fortunately, refs. [32,33] have shown that the worst-case crack growth equation associated with the growth of naturally occurring 3D cracks in BSI&WS LPBF Scalmalloy^®^ can be written as:

*da/dN* = 1.2 × 10^−10^ [(∆*K* − 0.1)/√(1 − *K*_max_/*A*)]^2^ (1)

Here Δ*K_thr_* is the fatigue threshold, and *A* is the apparent cyclic fracture toughness. The value of *A* used in [32,33], was 53 MPa √m. (Here it should be noted that there was a typing error in [32,33] where the value of *A* was incorrectly reported as 35 MPa √m.) These papers also revealed how, in accordance with the certification guidelines delineated in USAF Structures Bulletin EZ-SB-19-01 [5], Equation (1) could be used to predict the growth of naturally occurring 3D cracks with depths equal to or less than the mandated minimum size equivalent initial damage size (EIDS). To date [32,33] are the only studies that have shown how to predict the growth of naturally occurring 3D cracks in LPBF Scalmalloy^®^ where the crack sizes are consistent with the mandated minimum size EIDS.

Consequently, the crack growth predictions associated with these various tests were performed using Equation (1). The only unknown in this equation is the value of the cyclic fracture toughness (*A*). Fortunately, the Alcoa [51] study revealed that the apparent cyclic fracture toughness (*A*) for AA7085-T7452 would appear to be approximately 65 MPa √m. As such, the crack growth equation to be used in these (AA7085-T7452) predictions becomes:

*da/dN* = 1.2 × 10^−10^ [(∆*K* − 0.1)/√(1 − *K*_max_/65)]^2^ (2)

As in previous papers [32,33,54,55,56], it was assumed that the crack(s) can be represented as a part elliptical crack. Armed with this assumption, the value of the stress intensity factors around the crack front was computed using the 3D finite element alternating approach, and the change in the (three-dimensional) shape of the crack was computed using Equation (2). As explained in [32,33,54,55,56,57,58,59,60,61], an advantage of using the three-dimensional finite element alternating method is that the cracks are not modelled explicitly and, regardless of the shape of the crack, only the uncracked finite element model is needed. Detail discussions on the finite element alternating method can be found in [56,57,58,59,60,61].

Consequently, in order to determine the stress intensity factors for any given crack configuration, it was first necessary to develop a three-dimensional finite element model of the repaired structure. In this paper, the Young’s modulus and Poisson’s ratio of 7075-T7351 were taken to be 73,000 MPa and 0.3, respectively. Details of the meshes used in the analysis are given in the Sections related to the various test programs. For simplisticty the results of the various analyses will be discussed together with the results obtained for the various test prgrams. In other words the predictions associated with the test program 3, i.e., tests on AA7085-77452 specimens with a hole, will be presented in the Section 4

## 3. Results of the First Test Program—The Effect of Exposure on AA7085-T7452

**Figure 5:**
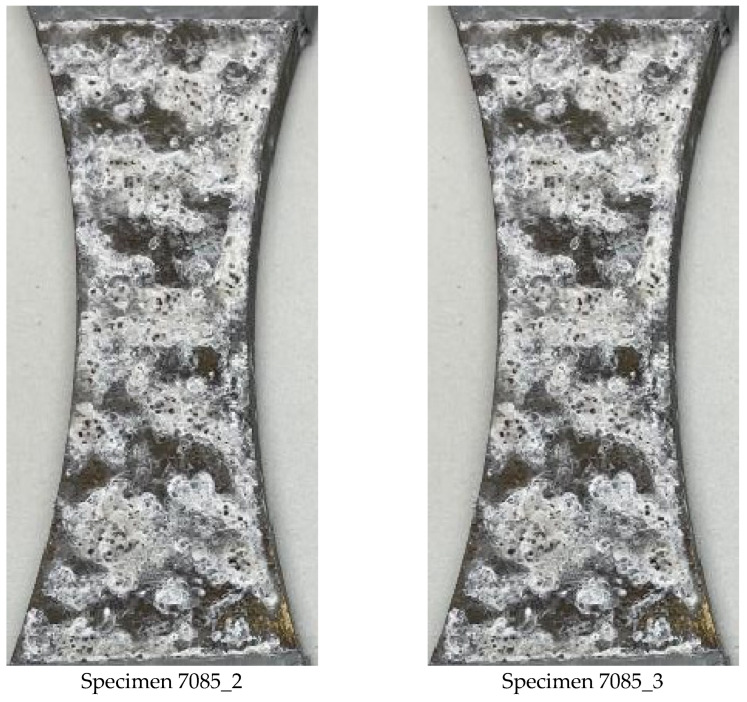
Photographs of the extensive corrosion seen by specimens 7085_2 and 7085_3 after fourteen days exposure to a 5 wt% NaCl salt fog at 35 °C.

Pictures of the extensive the corrosion seen by these AA7085-T7452 specimens, after fourteen days, are given in Figure 5. Here it should be noted that, although the BSI&WS LPBF AM Scalmalloy^®^ specimens were exposed (in the same environmental chamber) for twenty-eight days the extent of the corrosion damage seen by the AA7085-T7452 specimens was such that the exposure tests had to be stopped after only fourteen days.

### Preliminary Assessment of the Surface Topography

**Figure 6:**
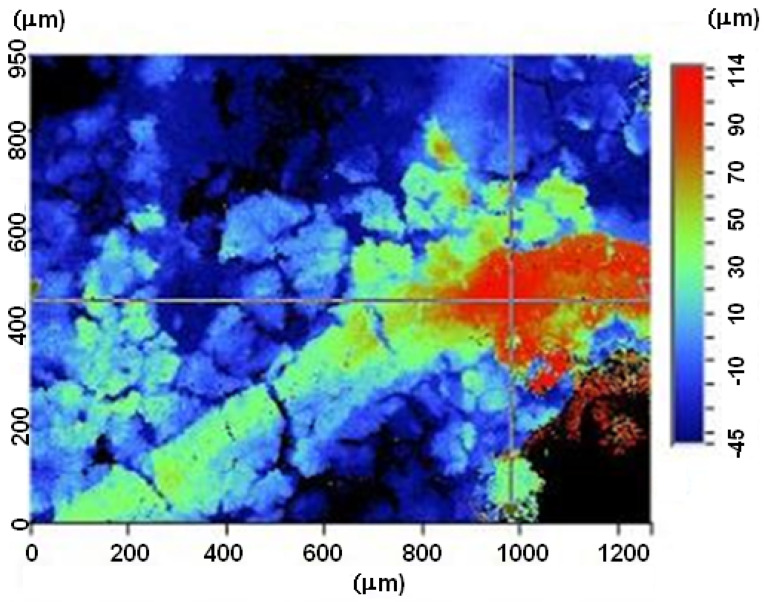
A typical local surface topography measurement—Specimen 7085_2, all units are in µm.

**Figure 7:**
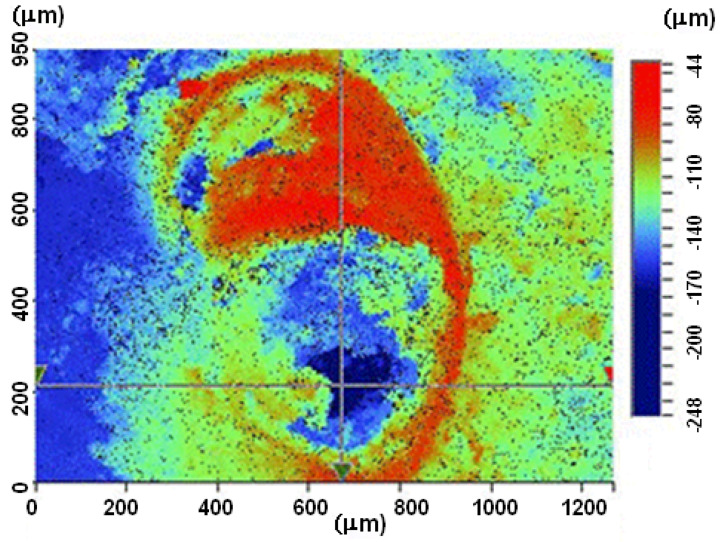
A typical local surface topography measurement—Specimen 7085_3.

Prior to fatigue testing, localised surface topography measurements were made in order to obtain approximate estimates of the heights of any protrusions and the depths of any pits. This was done using a 3D optical profiler (Bruker Contour GT, Tucson, AZ, USA) together with the analysis software WYKO Vision 32 and Profilm Online. The measurements obtained are shown in Figure 6 and Figure 7. Figure 7 reveals a loss of material of approximately 0.25 mm surface depth. (In contrast, as reported by Ang et al. [33], Scalmalloy^®^ specimens that were exposed to the same ASTM B117-19 5 wt% NaCl salt fog environment at 35 °C, albeit for twenty-eight days rather than the fourteen days in the current study, experienced no measurable loss of material.) However, subsequent fractographic measurement, that were taken post fatigue testing of these two AA 7085-T7452 specimens revealed, that these measurements underestimated the size of the corrosion pits.

The corrosion pit sizes encountered in this (AA7085-T7452) test program were somewhat larger than that given by Tao et al. [13], which reported pit depths of approximately 0.12 mm after twelve months outdoor exposure of AA7085-T7452 to a hot-humid marine environment at a location on Hainan Island, China. (The average air temperature, relative humidity, and Cl- deposition rate reported by Tao et al. [13] were approximately 23.9 °C, 87.6%, and 14.6 mg/(m^2^·d), respectively.) Shi et al. [14] reported similar (AA7085-T7452) corrosion depths, albeit after only three months exposure, at the same location. On the other hand, the corrosion depths are consistent with those reported by Barter et al. [49] and Main et al. [53] as well with the corrosion depths reported by McAdam et al. [18] for AA7085-T7452 specimens that had been exposed, under shelter, for twelve months at RAAF, RAAF Base Williamtown in Australia.

## 4. Results of the Fatigue Test Program on Pre-Exposed Base-Line AA7085-T7452 SPECIMENS

### 4.1. Fatigue Failure of Specimen 7085_2

**Figure 8:**
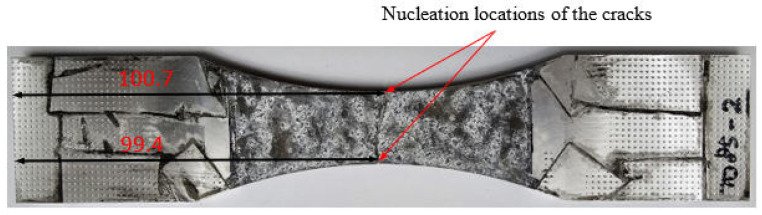
Plan view of the failure of Specimen 7085_2, the dimensions of the specimen are as per Figure 3.

**Figure 9:**
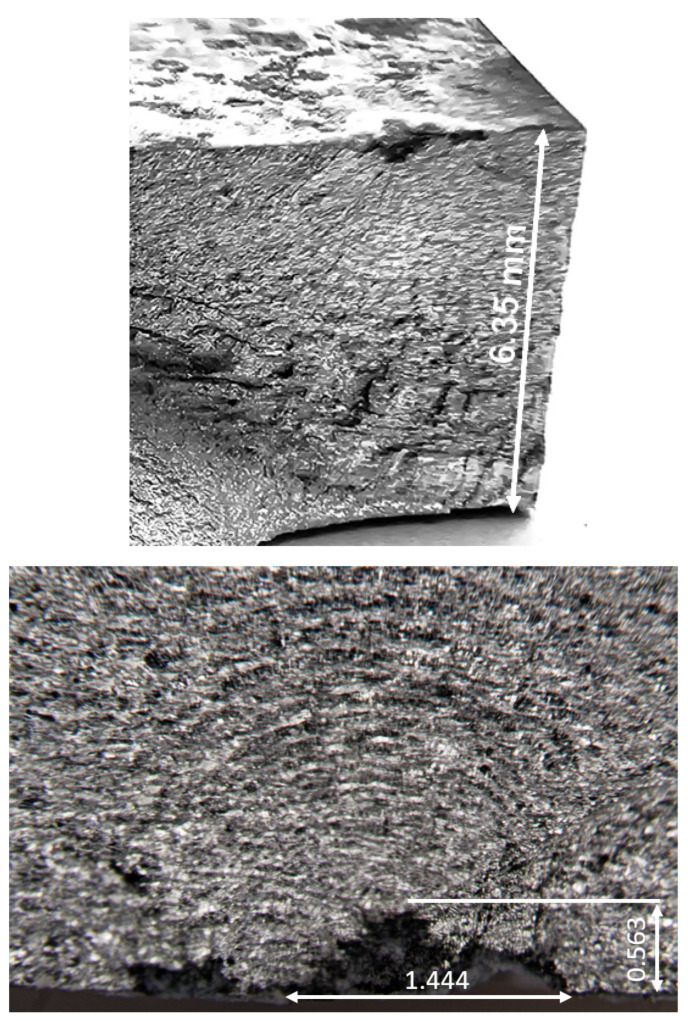
Views of the failure surface and a rotated view of the nucleating corrosion pit associated with specimen 7085_2, all dimensions are in mm.

**Figure 10:**
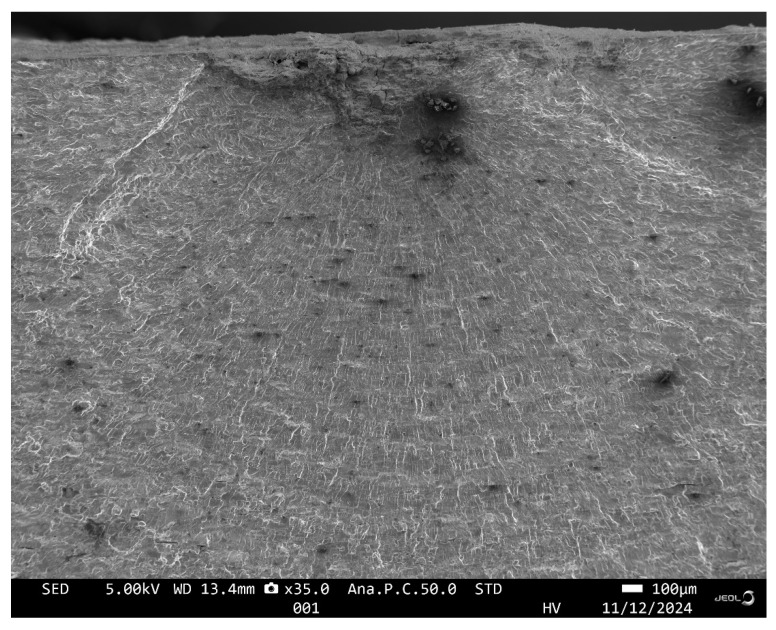
SEM of the locations associated with the primary (lead) crack in Specimen 7085_2.

Specimen 7085_2 failed after 573,916 cycles, which equates to approximately 55.72 marker blocks. A plan view of the failure is given in Figure 8, and pictures of the failure surface are given in Figure 9 and Figure 10. As can be seen in Figure 9 and Figure 10, failure occurred as a result of a dominant crack that nucleated at a corrosion pit that was approximately 0.56 mm deep and had a (tip to tip) surface length of approximately 1.44 mm. The dimensions associated with this pit are consistent with, albeit slightly worse than, the corrosion depths reported by McAdam et al. [18] for AA7085-T7452 specimens that had been exposed, under shelter, for twelve months at RAAF Base Williamtown in Australia. However, this pit depth is less than the maximum corrosion penetration depth of 1.2 mm reported by McAdam et al. [18] for an operational aircraft. The failure surface was essentially at 90° to the direction of the load.

### 4.2. Specimen 7085_3

**Figure 11:**
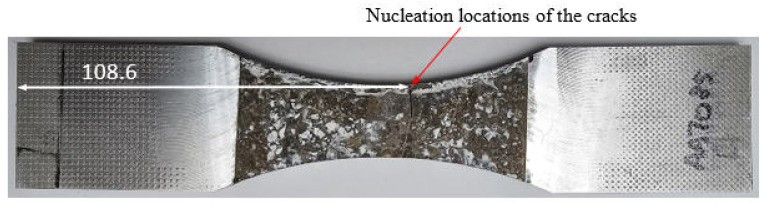
Plan view of Specimen 7085_3, all dimensions are in mm.

**Figure 12:**
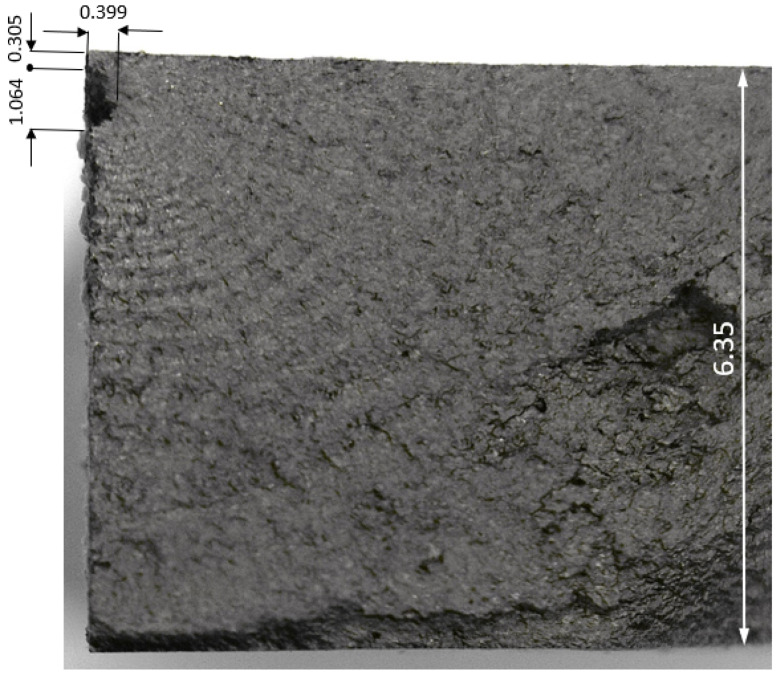
The failure and the failure surface associated with specimen 7085_3, all dimensions are in mm.

**Figure 13:**
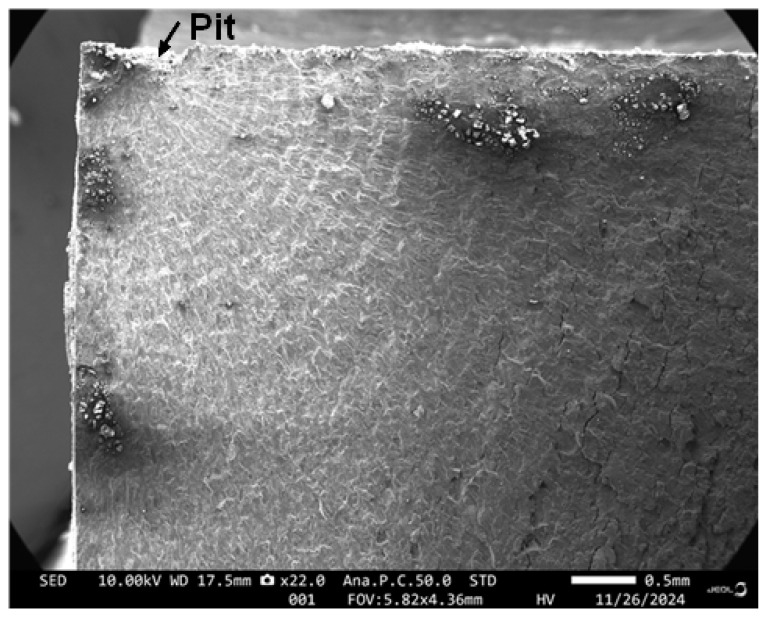
SEM pictures of the failure location, Specimen 7085_3. Note that in this picture the specimen has been rotated so that the image in Figure 10 is at 90° to that shown in Figure 9.

Specimen 7085_3 failed after 711,035 cycles, which equates to approximately 69.03 marker blocks. A plan view of the failure is given in Figure 11, and pictures of the failure surface are given in Figure 12 and Figure 13. As can be seen in Figure 12 and Figure 13, failure occurred as a result of a crack that nucleated at an approximately 0.40 mm deep and 1.06 mm wide corrosion pit. The dimensions associated with this pit are also consistent with, albeit slightly worse than, those reported the corrosion depths reported by McAdam et al. [18] for AA7085-T7452 specimens that had been exposed, under shelter, for twelve months at RAAF Base Williamtown in Australia.

## 5. Results of Predicting the Crack Growth Histories Seen in the Base-Line 7085-T7452 Test Program

It has long been known [46,62,63,64,65,66,67] that, with the exception of the region where the crack is close to failure, the growth of naturally occurring cracks in military aircraft is often approximately exponential. In other words, there is often a near-linear relationship between ln(*a*), where *a* is the crack depth, and the number of cycles (*N*).

**Figure 14:**
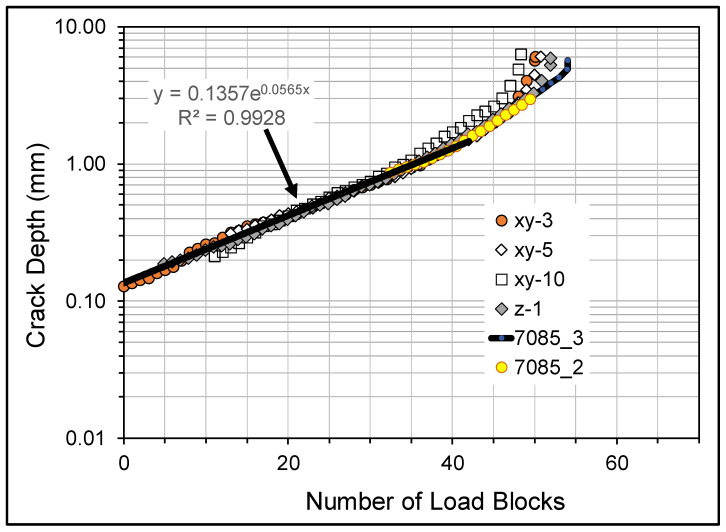
Comparison of 7085-T7452 specimens versus BSI&WS LPBF built Scalmalloy^®^ specimens.

Since the present test program used specimens with the same geometry and was tested using the same load spectrum as the BSI&WS LPBF built Scalmalloy^®^ specimens xy-3, xy-5, xy-10, and z-1, reported in [32,33], the crack growth histories in the linear section of the relationship between ln(*a*) and *N* for the BSI&WS LPBF built Scalmalloy^®^ specimens were compared with those of the AA 7085-T7452 specimens. As can be seen in Figure 14, which presents a plot of the crack growth histories for the Scalmalloy^®^ specimens post a crack depth of approximately 0.125 mm (1/8th inch) and the various AA7085-T7452 specimen crack growth curves, shifted so that at the first measurable crack size the various curves coincided with this Scalmalloy^®^ curve, the various curves are very similar. In other words, post the mandated minimum EIDS of 0.254 mm [4,5] the crack growth curves are similar.

Here it should be noted that whilst Scalmalloy^®^ specimen xy-5, which is reported in [32], was not exposed to an ASTM B117-19 Standard 5 wt% NaCl salt fog environment at 35 °C, prior to fatigue testing Scalmalloy^®^ specimens xy-3, xy-10, and z-1 were exposed to the same exposed to an ASTM B117-19 Standard 5 wt% NaCl salt fog environment at 35 °C as the 7085-T7452 specimens. However, whereas the 7085-T7452 specimens were only exposed for fourteen days the Scalmalloy^®^ specimens were exposed for twenty-eight days, see [33] for more details. As such, Figure 14 reinforces the statement given in [33] that prior exposure appeared to have little effect on the durability of BSI&WS LPBF built Scalmalloy^®^.

### 5.1. Predicting the Crack Growth History Associated with Specimen 7085_2

As in prior studies [32,33,54,55,56] into the growth of naturally occurring 3D cracks in AM materials, for each crack shape the stress intensity factor distribution around the crack was computed using the three-dimensional finite element alternating method. (The mathematics underpinning this approach is explained in [57,58,59,60,61]). Once the stress intensity factors around the circumference of the crack was computed, Equation (2) was then used to compute the increment in the crack growth, and hence the next crack shape. This analysis procedure was repeated until failure.

**Figure 15:**
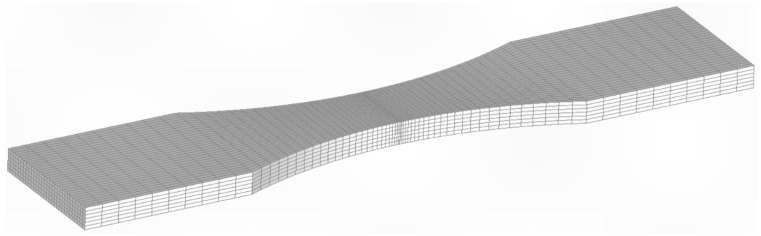
The fine mesh used in the crack growth predictions for both specimens 7085_2 and 7085_3.

As explained in Section 2, when using the finite element alternating approach to compute the stress intensity factor solution, for any given crack shape, it is only necessary to use a finite element model of the uncracked structure. In this study two different finite element meshes of the uncracked specimen were developed. The first, a coarse mesh, had 4160 twenty-noded iso-parametric elements and 20,777 nodes. The second, a finer mesh, had 12,600 iso-parametric elements and 58,713 nodes, see Figure 15. The stress field at the location where the crack nucleated in these two analyses differed by less than 1.5%. The crack growth analysis used stress intensity solutions determined using the fine mesh in conjunction with Equation (2).

**Figure 16:**
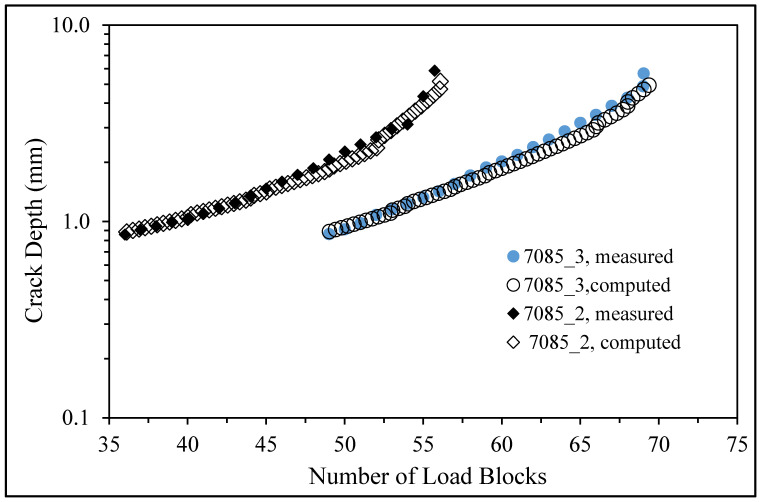
The measured and predicted crack depth histories associated with the lead crack in Specimens 7085_2 and 7085_3.

The initial size of the crack in the analysis of Specimen 7085_2 was taken from the first well-defined marker band, viz, a = 0.858 mm and c = 0.763 mm. Here, *a* is the crack depth as measured from the surface of the specimen, and *c* is the crack half-length (at the surface). The resultant measured and predicted crack depth histories are shown in Figure 16, where we see good agreement. 

### 5.2. Predicting the Crack Growth History Associated with Specimen 7085_3

The crack growth prediction for specimen 7085_3 also used the fine mesh shown in Figure 14. In this instance, the stress field at the location where the crack nucleated in the two analyses was found to differ by less than 1.3%. However, for specimen 7085_3, the initial size of the crack in the analysis, which was taken from the first well-defined marker band, was *a* = 0.863 mm and *c* = 0.869 mm. (As previously mentioned, a is the crack depth as measured from the upper surface of the specimen and c is the crack half-width.) The resultant measured and computed crack depth histories are also shown in Figure 15, where we again see good agreement.

These findings support the conclusion that the growth of naturally occurring 3D cracks in BSI&WS LPBF built Scalmalloy^®^ and AA 7085-T7452 is similar. This finding, when coupled with the realisation that both materials have similar mechanical properties, highlights Scalmalloy’s^®^ potential to be used to rapidly print drones and attritable aircraft. On the other hand, these findings, when coupled with the fact that BSI&WS LPBF built Scalmalloy^®^ would appear to be more resistant to corrosion pitting than AA7085-T7452, further highlights its potential/suitability to be used to build AM replacement parts for aluminium alloy aircraft that are operated in an aggressive maritime environment.

## 6. Results of the Third AA7085-T7452 Test Program—A Specimen with a ¼ Inch (6.35 mm) Diameter Fastener Hole

**Figure 17:**
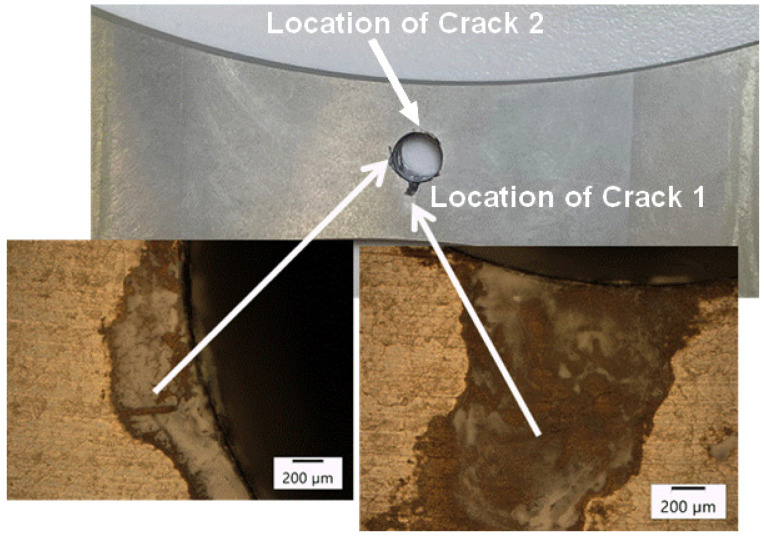
Corrosion damage at the intersection of the bore of the hole and the annodised surface.

As can be seen in Figure 17, after fourteen days of exposure the specimen had corrosion damage at the intersection of the bore of the hole and the anodised surface. The depth of the damage was approximately 0.1 mm.

As explained in Section 2, the load spectrum applied to this specimen consisted of three different repeated marker load blocks. In each of these repeated load blocks the load is held constant and the spectrum consisted of 15,000 cycles at *R* = 0.8 and 300 cycles at *R* = 0.1. The first spectrum had a max load in the spectrum of 10.8 kN and was used to sharpen the frack. Once the surface crack length associated with Crack 1 had reached approximately 0.34 mm Spectrum 2 was used. The maximum load in Spectrum 2 was 13.0 kN. Spectrum 2 was used until the surface crack length associated with Crack 1 reached a length of approximately 1.5 mm. At this stage the maximum load in the spectrum was increased to 15.17 kN. Specimen A-7085-1 failed after experiencing 210,419 cycles of Spectrum 3.

**Figure 18:**
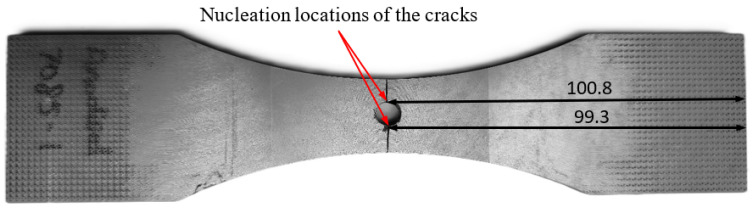
A plan view of the failed specimen, all dimensions are in mm.

**Figure 19:**
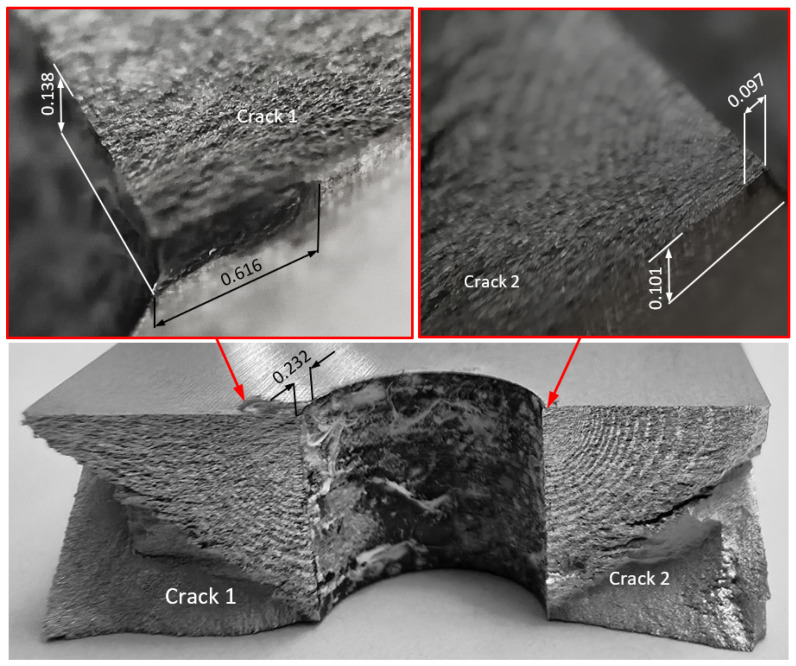
The failure surfaces associated with specimen A-7085-1, all dimensions are in mm.

**Figure 20:**
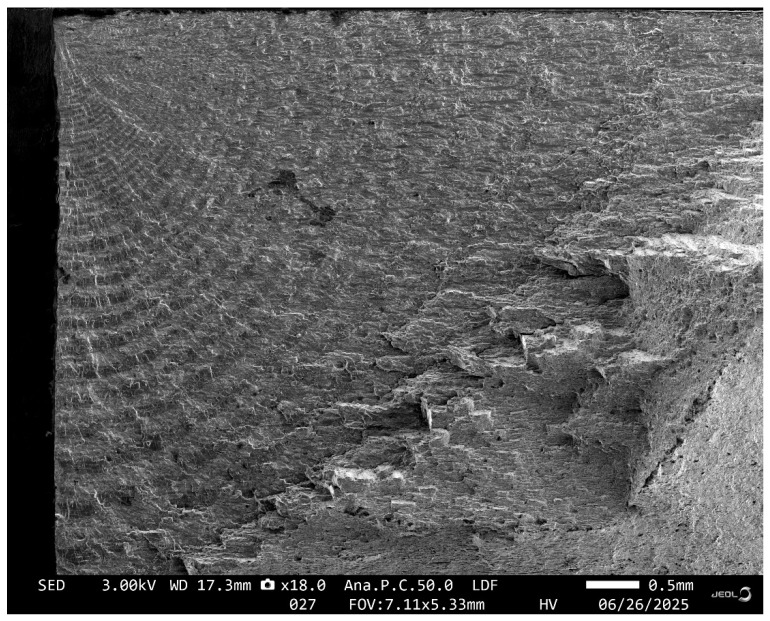
SEM for the Crack 1 in specimen A-7085-1.

**Figure 21:**
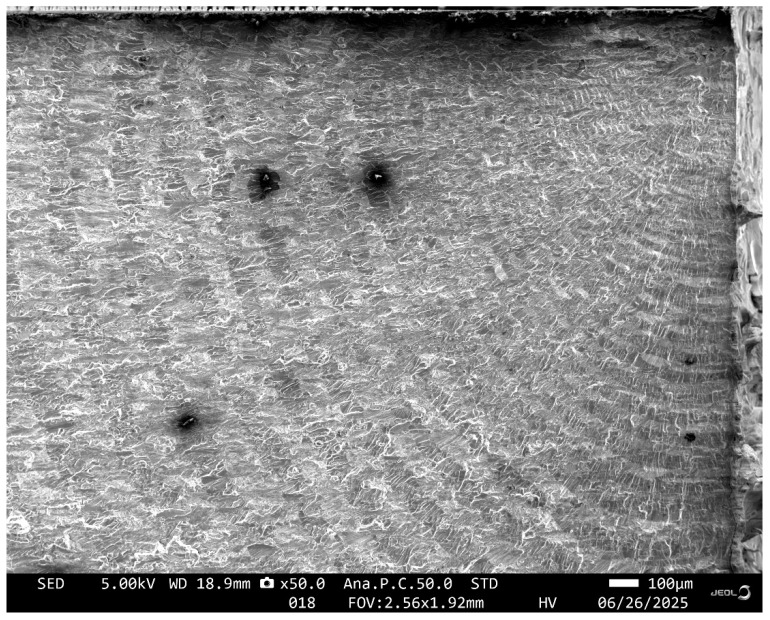
SEM for the Crack 2 in specimen A-7085-1.

A plan view of the failed specimen is shown in Figure 18. A cross sectional view of the failure is shown in Figure 19. Here we see that failure was due to two diametrically opposed cracks that nucleated, at the centre line of the specimen, at the intersection between the anodised surface and the bore of the hole. These cracks were termed Crack 1 and Crack 2. SEM pictures of these two cracks are shown in Figure 20 and Figure 21.

### Predicting the Crack Growth History Associated with Specimen A-7085-1

**Figure 22:**
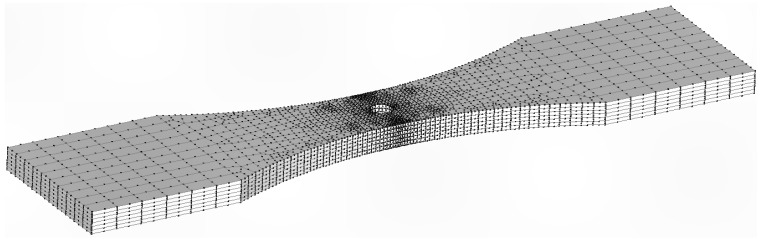
Mesh 1: 4980 twenty nodded iso-parametric tetrahedral elements and 27,930 nodes.

**Figure 23:**
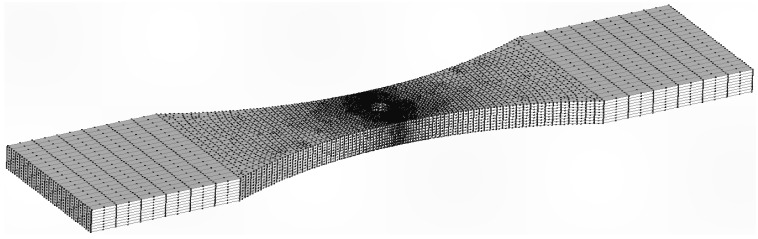
Mesh 2: 16,832 twenty nodded iso-parametric tetrahedral elements and 76,708 nodes.

**Figure 24:**
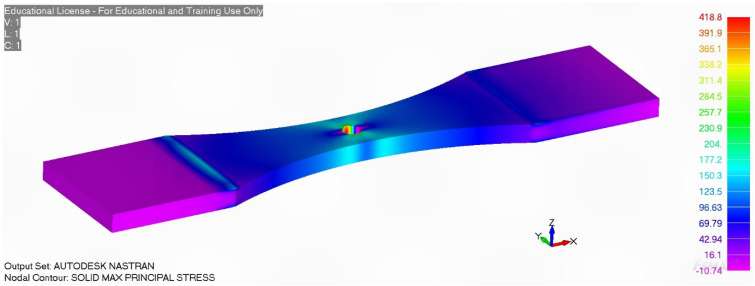
The maximum principal stress for Specimen A-7085-1 at a remote load of 15.169 kN, the stress units are in MPa. The stress field at the location where the crack(s) nucleated in the two analyses was found to differ by less than 1.4%.

As previously two different finite element meshes of the uncracked specimen were developed. The first, a coarse mesh, had 4980 twenty-noded iso-parametric elements and 27,930 nodes. The second, which was a finer mesh, had 16,822 twenty-noded iso-parametric elements and 76,708 nodes, see Figure 22 and Figure 23. The stress field at the location where the crack nucleated in these two analyses differed by less than 1.4%, see Figure 24. The crack growth analysis used stress intensity solutions determined using the fine mesh in conjunction with Equation (2).

**Figure 25:**
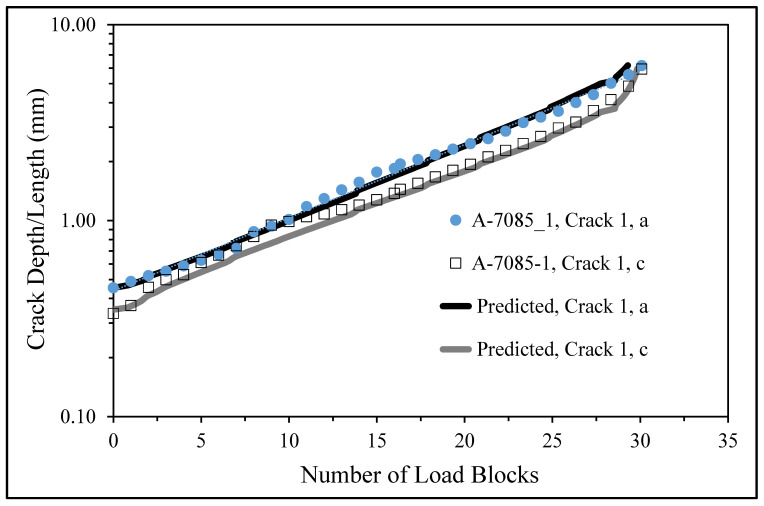
The measured and computed curves, post the initial crack lengths a_0_ and c_0_, associated with the Crack 1 in Specimen A-7085-1.

**Figure 26:**
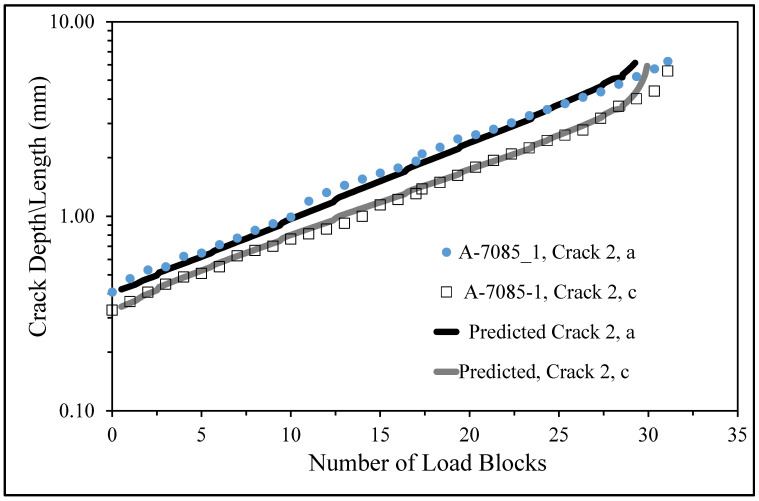
The measured and computed curves, post the initial crack lengths a_0_ and c_0_, associated with the Crack 2 in Specimen A-7085-1.

The measured and predicted cracked growth histories for Crack 1 post a crack size of a_0_ = 0.4538 mm and c_0_ = 0.3359 mm, where a is the crack depth and c is the crack surface length are shown in Figure 25. The measured and predicted cracked growth histories for Crack 2 post a crack size of a_0_ = 0.4074 mm and c_0_ = 0.329 mm, where a is the crack depth and c is the crack surface length are shown in Figure 26. Unfortunately, as can be seen in Figure 21 and Figure 22, the crack growth histories could only be accurately determined post these values of a_0_ and c_0_. There were approximately 31 load blocks from this size crack to failure.

The good agreement shown in Figure 25 and Figure 26 between the measured and predicted crack growth histories, for both Crack 1 and Crack 2, further supports the result reported in Section 5 that the equation governing the growth of small naturally occurring cracks in AA7085-T7452 can be expressed as per Equation (2). In other words, that the crack growth equation associated with the growth of 3D cracks from surface corrosion damage is similar to the crack growth equation associated with the growth of naturally occurring cracks in Scalmalloy^®^. As such, Figure 25 and Figure 26 also support the prior findings reported in Section 4 that the growth of naturally occurring 3D cracks in Scalmalloy^®^ and in pre-corroded 7085-T7452 is similar and that crack growth can be computed using the same equation developed for the growth of naturally occurring 3D cracks in BSI&WS LPBF built Scalmalloy^®^, albeit with an allowance made for the difference in the cyclic fracture toughness.

## 7. Discussion—Implications for AM Scalmalloy

This paper is the first to show that BSI&WS LPBF Scalmalloy^®^ not only has similar mechanical properties to that of conventionally built AA7085-T7452, but that it also has a similar crack growth equation. However, as previously mentioned, both MIL-STD-1530Dc [4] and USAF Structures Bulletin EZ-SB-19-01 [5] require a durability assessment for limited-life AM replacement parts. The guidelines for performing this durability assessment are given in [66] and summarised by Yang et al. in [67]. Yang et al. [67] explain how this can be achieved if it can be shown that crack growth can be expressed in the form:

*a* = *a_EIDS_* e^(*Qt*)^ (3)

where *a* is the crack size, *t* is the flight time (or number of cycles or load blocks), *Q* is a spectrum dependent parameter, and *a_EIDS_* is the back projected crack size at *t* = 0. (In [4,5] the term *a_EIDS_* is referred to as the equivalent initial damage size.) This formulation also forms the basis for the USAF approach to assessing the risk of failure [62]. MIL-STD-1530Dc [4] and USAF Structures Bulletin EZ-SB-19-01 [5] also suggest that the DADT assessment of an AM part use linear elastic fracture mechanics (LEFM).

It should also be recalled that is now known that:

(a) the growth of naturally occurring 3D cracks in BSI&WS LPBF Scalmalloy^®^ can be predicted using LEFM [32,33];
(b) the growth of naturally occurring 3D cracks in BSI&WS LPBF Scalmalloy^®^ conforms to Equation (3);
(c) Scalmalloy^®^ has a superior damage tolerance than conventionally built AA7075-T6 [31];
(d) Scalmalloy^®^ has mechanical properties that are comparable with conventionally built AA7050-T7541 and AA7085-T7452;
(e) Scalmalloy^®^ is significantly more resistant to corrosion pitting than AA 7085-T7452;

These observations point to BSI&WS LPBF Scalmalloy^®^ being an ideal candidate for building limited-life parts for military aircraft. It also suggests that BSI&WS LPBF Scalmalloy^®^ is an ideal candidate for building drones and attritable aircraft. Furthermore, its documented resistance to corrosion pitting has the potential to reduce maintenance costs and thereby increase aircraft availability. This potential would be enhanced if the proposed NASA [39] approach to the certification of AM parts, which is based on ‘material equivalence’, is adopted.

## 8. Conclusions

Although the mechanical properties of both Scalmalloy^®^ and AA7085-T7452 have previously been (separately) reported in the open literature, this paper is the first to reveal that Scalmalloy^®^ has a yield stress, ultimate strength and elongation to failure that are similar to those of AA 7085-T7452. It is next shown that (unprotected) AA7085-T7452 specimens, i.e., specimens that do not have a corrosion protection coating, exposed to an ASTM B117-19 standard 5 wt% NaCl salt fog at 35 °C for fourteen days resulted in extensive corrosion with pits that could be up to approximately 0.5 mm deep. It is also reported that this size of these corrosion pits is broadly consistent with those seen by AA 7050-T7451 parts on RAAF F/A-18 Classic Hornet airframes that had experienced extended periods of downtime. This susceptibility (of AA 7085-T7452) to corrosion pitting contrasts with that seen in prior tests on unprotected Scalmalloy^®^ that, when exposed to the same environment in the same ASTM B117-19 environmental chamber for twenty-eight days, experienced minimal corrosion damage.

It is subsequently shown that the crack depth versus cycles histories associated with cracks that nucleated in these baseline pre-corroded AA 7085-T7452 specimens, i.e., specimens without a fastener hole, would appear to be similar to that associated with the growth of small cracks in BSI&WS LPBF built Scalmalloy^®^. (Three of the Scalmalloy^®^ test specimens considered had been in the same environment, albeit for twenty-eight days, and one specimen had not been exposed.) This observation led to the realization hat the crack growth equation governing the growth of naturally occurring 3D cracks in BSI&WS LPBF built Scalmalloy^®^ could be used to reasonably accurately predict the crack growth histories in these pre-corroded AA 7085-T7452 specimen tests. This finding was subsequently supported by tests on an anodised 7085-T7452 specimen with a fastener hole that had also been exposed to the same environment for fourteen days where it is shown that the crack growth histories associated with naturally occurring cracks can be accurately predicted using the same crack growth equation, i.e., Equation (2).

The fact that Scalmalloy^®^ and AA 7085-T7452 have

(i) similar mechanical properties;
(ii) that naturally occurring 3D cracks in BSI&WS LPBF built Scalmalloy^®^ and pre-corroded AA7085-T7452 have similar crack growth rates, and similar crack growth equations;
(iii) that Scalmalloy^®^ is significantly more resistant to corrosion pitting than AA 7085-T7452;

When taken together with the prior studies, which have compared BSI&WS LPBF built Scalmalloy^®^ to AA 7075-T6 and AA 2024-T3, further reinforces the suggestion that Scalmalloy^®^ may be suitable for use as limited-life replacement parts for corroded aircraft components. Furthermore, its documented resistance to corrosion pitting has the potential to reduce maintenance costs and thereby increase aircraft availability.

## Data Availability

The original contributions presented in this study are included in the article. Further inquiries can be directed to the corresponding author.

## Nomenclature

a	crack, length
a_0_	initial crack depth
*A*	cyclic fracture toughness
AA	aluminium alloy
AM	Additively manufactured
ASTM	American Society for Testing and Materials
*da/dN*	rate of fatigue crack (i.e., delamination) growth (FCG) per cycle
*BSI&WS*	Boeing Space, Intelligence and Weapon Systems
c_0_	initial crack (surface) length
DADT	durability and damage tolerance
EAC	environmentally assisted cracking
EASA	European Aviation Safety Authority
EIDS	equivalent initial damage size
FCG	fatigue crack growth
K	stress-intensity factor
$K_{max}$	maximum value of the applied stress-intensity factor in the fatigue cycle
$K_{min}$	minimum value of the applied stress-intensity factor in the fatigue cycle
∆K	$=K_{max}-K_{min}$
LEFM	linear-elastic fracture-mechanics
LPB	laser powder bed fusion
*N*	number of fatigue cycles
*NASA*	North American Space Administration
*P_max_*	maximum load applied during the fatigue test
*P_min_*	minimum load applied during the fatigue test
R	load ratio (=*P_min_*/*P_max_*)
RAAF	Royal Australian Air Force
SAE	Society of Automotive Engineers
SEM	scanning electron microscope
US	United States
USAF	United States Air Force
US DoD	United States Department of Defense
σ_y_,σ_ult_	Yield stress and ultimate strength
3D	three dimensional
